# Eveningness intensifies the association between musculoskeletal pain and health-related quality of life: a Northern Finland Birth Cohort Study 1966

**DOI:** 10.1097/j.pain.0000000000002609

**Published:** 2022-02-07

**Authors:** Eveliina Heikkala, Markus Paananen, Ilona Merikanto, Jaro Karppinen, Petteri Oura

**Affiliations:** a Center for Life Course Health Research, University of Oulu, Oulu, Finland; b Medical Research Center Oulu, University of Oulu and Oulu University Hospital, Finland; c Rovaniemi Health Center, Rovaniemi, Finland; d Primary Health Care Services, City of Espoo, Espoo, Finland; e SleepWell Research Program, Faculty of Medicine, University of Helsinki, Helsinki, Finland; f Department of Public Health Solutions, Finnish Institute for Health and Welfare, Helsinki, Finland; g Orton Orthopaedics Hospital, Helsinki, Finland; h Rehabilitation Services of South Karelia Social and Health Care District, Lappeenranta, Finland; i Department of Forensic Medicine, Faculty of Medicine, University of Helsinki, Helsinki, Finland; j Forensic Medicine Unit, Finnish Institute for Health and Welfare, Helsinki, Finland

**Keywords:** Musculoskeletal pain, Chronotype, Health-related quality of life, Cohort study

## Abstract

People with an evening (E)-type preference (ie, chronotype) experience musculoskeletal (MSK) pain and reduced health-related quality of life (HRQoL) more often than morning (M) types. Musculoskeletal pain is a well-established contributor to reduced HRQoL. This study aimed to evaluate whether eveningness amplifies the association between MSK pain and HRQoL in contrast to morningness. Questionnaire data on MSK pain dimensions (intensity, disability at work, number of pain sites [NPSs], and frequency), chronotype, covariates (sex, sufficiency of sleep duration, mental distress, and presence of coexisting diseases), and HRQoL (measured by 15D) were collected among 46-year-old individuals belonging to the Northern Finland Birth Cohort 1966 (N = 4257). Individuals without any MSK pain were excluded. General linear models were conducted to estimate the associations between chronotypes, MSK pain dimensions, and HRQoL. The interaction terms (chronotype × pain dimension) were tested in the models. There were 13% E-types and 43% M-types in the study sample. Each pain dimension and chronotype were related to HRQoL. In the sex-adjusted chronotype-specific models, the reduction in HRQoL in relation to pain appeared to be stronger among E-types than among M-types in respect to all pain dimensions. After adjustments, this was particularly seen in terms of NPS and pain frequency. Our findings suggest that eveningness intensifies the association between MSK pain and HRQoL, and, thus, they are indicative of E-types being more sensitive than M-types to the consequences of MSK pain. As such, MSK pain treatment and rehabilitation actions to improve HRQoL should be especially targeted at E-types.

## Abstract 2

The associations between 4 pain dimensions and health-related quality of life were stronger among evening than morning chronotypes with musculoskeletal pain.

Supplemental Digital Content is Available in the Text.

## 1. Introduction

Musculoskeletal (MSK) pain is a typical health problem of our time. It ranges in etiology from nonspecific (eg, nonspecific low back pain) to specific (eg, osteoarthritis) and is highly prevalent, especially among middle-aged populations.^22,23^ According to the prevailing view, MSK pain occurs in a multidimensional context in which biological, psychological, and social processes interact.^20^

Musculoskeletal pain is a highly disabling condition that significantly reduces health-related quality of life (HRQoL) by, for instance, limiting daily life function^8^ and participation in social life.^2^ In 2016, low back pain by itself accounted for 57 million years lived with disability worldwide.^17^ It has been estimated that more than half of the people living with pain in the general population report reduced HRQoL, compared with one-fifth (or less) of the pain-free population.^16,29^ At worst, people living with pain experience similarly low levels of HRQoL to palliative cancer patients.^15,50^ Because reduced HRQoL remains at a low level rather than improves over time,^19^ it is important to characterize the factors exposing individuals to this outcome. A reduction in HRQoL may be mitigated by identifying and accounting for the potential determinants.

Circadian rhythms reflect the 24-hour physiological and behavioral cycles within each individual.^11^ Based on individual variation in the timing of these innate rhythms, individuals can be categorized into 3 phenotypes (ie, chronotypes): *morning* (M—alertness level at its highest in the morning), *evening* (E—most active in the evening), and *intermediate* (I—neither M nor E). The chronotype is explained both by genetic and environmental factors^4^ and can be regarded as quite a robust attribute throughout adulthood.^5,9^

Evening types typically face more disruptions in their biological rhythms, for example, in their sleep–wake rhythm, than others. This is due to the early bedtimes and awakenings often required by working life.^34^ These disruptions are likely to expose E-types to adverse health-related behaviors and health problems,^6^ including MSK pain. Previously, E-types have been reported to be one-and-a-half times more likely to experience back pain and nearly twice as likely to require hospitalization because of back pain than M-types.^31,33^ Consequently, E-types also tend to have reduced HRQoL.^45,48^

To date, only a few studies have explored the significance of chronotype in pain-related HRQoL, with the existing evidence being conflicting and highly limited to nongeneral populations and small sample sizes.^18,48^ Further understanding of the potential role of chronotype in the MSK pain–HRQoL associations might help in designing personalized MSK pain treatment and rehabilitation measures to maintain or improve HRQoL,^27^ for instance, related to prognostic stratification of individuals with MSK.^10,13^ Therefore, in this study, we aimed to investigate whether the association between MSK pain and HRQoL is stronger among E-types than among M-types. This was done using a set of MSK pain dimension variables (pain intensity, pain-related disability at work, number of pain sites [NPSs], and pain frequency) with a large general population sample of middle-aged Finns from the Northern Finland Birth Cohort 1966 (NFBC1966).

## 2. Methods

### 2.1. Study population

**Figure 1.**
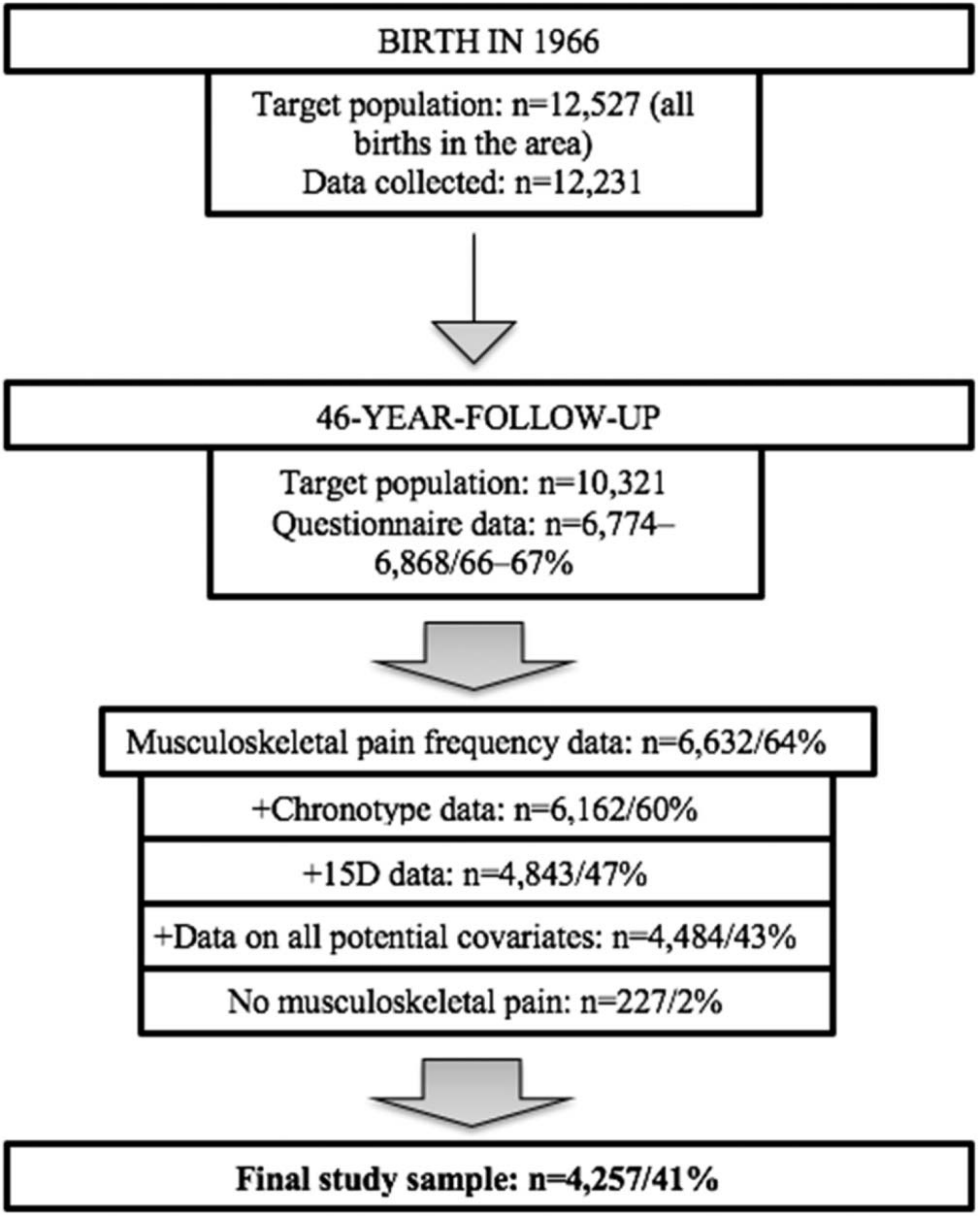
Flowchart of the data collection of the study sample.

The study sample comprises subjects who are part of a large and representative birth cohort of children born in 1966 (NFBC1966; Fig. 1). The cohort base originated from pregnant women living in the Northernmost provinces of Finland (Oulu, Lapland) and whose estimated gestation time suggested a delivery date between January 1, 1966, and December 1, 1966.^49^ The cohort recruits comprised 12,231 children (96% of all births), and the NFBC1966 members have been regularly monitored until the year of their 46th birthday. The present study focused on this most recent follow-up point, during which the participants were contacted via a package of questionnaires (response rate 66%-67% [n = 6774-6868], depending on the questionnaire). In addition to those who did not give permission to use their data, those with missing data on chronotypes, presence of MSK pain, and potential covariates were excluded. An additional 227 NFBC1966 members reported no MSK pain and, thus, were also excluded from the analyses. This left a final sample size of 4257 individuals (41% of the target population; Fig. 1). The study was approved by the Ethics committee of the Northern Finland Hospital District.

### 2.2. Health-related quality of life

Health-related quality of life was measured by 15D, a standardized and validated method for estimating HRQoL in general^42^ and in pain populations.^51^ 15D is constructed by 15 dimensions: mobility, vision, hearing, breathing, sleeping, eating, speech, excretion, usual activities, mental function, discomfort and symptoms, depression, distress, vitality, and sexual activity. In each dimension, individuals choose a response from 5 potential options (no, slight, considerable, severe, or unbearable problems), depending on the severity level that best describes their current health state. The total 15D score, ranging from 0 to 1 (0 = dead, 1 = full HRQoL), is calculated from a set of population-based utility or preference weights. More detailed information on the valuation system used has been provided elsewhere.^43^ The minimum clinically important change in the total 15D score (ie, an individually identifiable improvement or reduction in the 15D score) has been reported to be 0.015.^1^

### 2.3. Musculoskeletal pain

The questionnaire asked about the presence or absence of MSK pain within the preceding year as follows: “Have you had any pain or ache in the following body parts within the last 12 months? (1) neck, (2) shoulder, (3) arms/elbows, (4) wrists/hands, (5) low back, (6) hips, (7) knees, and (8) ankles/feet.” The response options were (1) no, (2) on 1 to 7 days, (3) on 8 to 30 days, (4) on more than 30 days but not daily, and (5) daily. All pain locations were combined to form a variable in which the overall frequency of pain was determined as the highest reported frequency category for any of the 8 body locations. The frequency was then grouped as (1) 1 to 7 days, (2) 8 to 30 days, (3) more than 30 days, and (4) daily, depending on the responses to the pain question. The first frequency category was regarded as a reference. Pain-related intensity and disability at work were based on the question: “If you have had musculoskeletal pain within the preceding year, how intense and disabling have you experienced the pain to be?” Subjects reported overall intensity and disability at work using a numerical rating scale from 0 to 10 (0 = no pain, 10 = extremely intense pain/total disability). A NPSs variable was formed from the sum of all pain locations (range 1-8 sites). All pain dimension variables apart from frequency were considered as continuous variables.

### 2.4. Chronotypes

To assess chronotypes, we used a short version of the Morningness-Eveningness Questionnaire (sMEQ), containing 6 of the original MEQ items.^21^ In the sMEQ, individuals rated themselves in each item, on scales ranging from 0 to 6 (maximum total score = 27). Those who summed 5-12 points were regarded as E-types, 13-18 as I-types, and 19 to 27 as M-types, as recommended.^30^ In general, the MEQ has been shown to correlate with sleep–wake rhythm^12^ and the sMEQ as explaining 83% of the variance in the total MEQ score.^24^ In the Finnish population, the internal consistency of sMEQ has also been shown to be good,^30^ and the sMEQ has been shown to correlate with both free day and workday sleep–wake rhythms and a genetic tendency for morningness/eveningness.^35^

### 2.5. Covariates

The following variables were considered to be covariate candidates: sex,^16^ sufficiency of sleep duration,^16^ mental distress,^19,25^ and presence of coexisting diseases.^19^ These variables were constructed on the basis of the 46-year questionnaire.

The questionnaire asked NFBC1966 members to estimate any deviation in the sufficiency of sleep duration that had occurred at least 3 times per week within the last month and to evaluate whether sleep duration was (1) sufficient, (2) somewhat insufficient, (3) explicitly insufficient, or (4) completely insufficient.

The presence of mental distress was evaluated by Hopkins Symptom Checklist-25, which surveys depression and anxiety symptoms with 25 items scaled 1 to 4 (1 = not at all, 4 = extremely). The mean total score from all answers was calculated and then divided into 2 categories based on the previous recommendations^52^: 1.55 or above (“severe”) vs under 1.55 (“mild”). In the literature, Hopkins Symptom Checklist-25 has been recognized as a potential screening tool for psychiatric disorders.^52^

In the questionnaire, subjects provided data on 75 diseases, symptoms, and traumas for which they had received a diagnosis from a medical doctor. Of these, diseases that were significantly associated with HRQoL and were nontraumatic and chronic by nature were included (excluding mental health problems and MSK diseases) (Supplement 1, available as supplemental digital content at http://links.lww.com/PAIN/B584). As we were interested in the potential confounding effect of any coexisting disease on the relationships between pain dimensions and HRQoL, we dichotomized their presence as no vs yes.

In addition to potential covariate candidates, we estimated the prevalence of taking daytime naps and reported use of pain and sleep medication among the study participants. Taking daytime naps was indirectly measured by calculating the difference between self-reported daily sleep and nightly sleep and then dichotomizing the difference as yes (>0) vs no (=0). The following pain medication data, based on Anatomical Therapeutic Chemical codes, were included in the study: nonsteroidal anti-inflammatory medicines (M01A), paracetamol (N02BE01, N02BE51), neuropathic pain medicines (gabapentin N03AX12, pregabalin N03AX16, amitriptyline N06AA09, nortriptyline N06AA10, venlafaxine N06AX16, duloxetine N06AX21), and opioids (N02A). The selection of these medication data was based on the latest national guideline of pain.^37^ Sleep medication consisted of sleep medication coded as N05C, including, for example, nonbenzodiazepine sedatives. Both medication variables were dichotomized as yes vs no.

### 2.6. Statistical analysis

Categorical confounding and MSK pain variables were described by frequencies and percentages and compared between chronotypes by χ^2^ test. The mean values and SDs were presented for continuous variables, and the mean differences were tested via a Kruskal–Wallis test. The associations between (1) covariate candidates and HRQoL and (2) pain dimensions, chronotypes, and HRQoL were studied using general linear models, with beta coefficients (B) and their 95% confidence intervals (CIs). To analyze the potential strength discrepancy between chronotypes in the associations between pain dimensions and HRQoL, an interaction term for chronotype and each pain dimension (eg, chronotype × pain intensity) was included in the corresponding pain dimension-HRQoL model. The statistical significance of the interaction terms was interpreted in such a way that the association between pain and HRQoL was different, depending on chronotype. All the covariate candidates were significantly related to HRQoL in the univariate analyses and were, thus, incorporated in the adjusted models. To analyze the potential confounding effects of different covariates on the pain dimension-HRQoL associations, 3 adjusted models were constructed: model I was adjusted for sex only; model II additionally for mental distress and presence of coexisting diseases; and model III additionally for sufficiency of sleep duration. To identify selection bias related to nonrespondents, we compared the characteristics of the study sample and nonrespondents. Statistical analyses were performed using SPSS (version 27.0). The *P* value of <0.05 was deemed statistically significant.

## 3. Results

Table 1 presents the characteristics of the study population. Because of the low number of subjects in the explicitly insufficient and completely insufficient categories of the sufficiency of sleep duration variable, they were combined as “insufficient.” As for chronotype, a majority of the participants were I-types (n = 1878 [44%]) or M-types (1845 [43%]). A higher percentage of E-types than I- or M-types reported insufficient sleep duration (132 [25%] vs 180 [10%] and 84 [5%], respectively, *P* < 0.001), suffered from severe mental distress (186 [35%] vs 418 [22%] and 259 [14%], respectively, *P* < 0.001), had coexisting diseases (355 [67%] vs 1085 [58%] and 993 [54%], respectively, *P* < 0.001), and used sleep medication (44 [10%] vs 61 [4%] and 37 [2%], respectively, *P* < 0.001).

**Table 1:** Characteristics of the 46-year-old Northern Finland Birth Cohort 1966 members with musculoskeletal pain, stratified by chronotype.

Variables	Chronotype				*P*
	Evening (n = 534)	Intermediate (n = 1878)	Morning (n = 1845)	Total (n = 4257)	
Sex,* % (n)					0.016
Men	37 (195)	44 (816)	42 (774)	42 (1785)	
Women	63 (339)	56 (1062)	58 (1071)	58 (2472)	
Sufficiency of sleep duration,* % (n)					<0.001
Insufficient	25 (132)	10 (180)	5 (84)	9 (396)	
Somewhat insufficient	55 (292)	53 (1004)	39 (727)	48 (2023)	
Sufficient	20 (110)	37 (694)	56 (1034)	43 (1838)	
Sleeping naps,* % (n)					0.519
No	63 (310)	61 (1053)	62 (1046)	61 (2409)	
Yes	37 (183)	39 (687)	38 (640)	39 (1510)	
Mental distress,* % (n)					<0.001
Severe	35 (186)	22 (418)	14 (259)	20 (863)	
Mild	65 (348)	78 (1460)	86 (1586)	80 (3394)	
Presence of coexisting diseases,* % (n)					<0.001
Yes	67 (355)	58 (1085)	54 (993)	57 (2433)	
No	33 (179)	42 (793)	46 (852)	43 (1824)	
Using pain medication,* % (n)					0.352
Yes	65 (291)	69 (1065)	68 (1009)	68 (2365)	
No	35 (157)	31 (489)	32 (473)	32 (1119)	
Using sleep medication,* % (n)					<0.001
Yes	10 (44)	4 (61)	2 (37)	4 (142)	
No	90 (404)	96 (1493)	98 (1445)	96 (3342)	
Pain intensity,† mean (SD)	4.5 (2.6)	4.2 (2.5)	4.0 (2.6)	4.1 (2.6)	<0.001
Pain-related disability at work,† mean (SD)	4.0 (2.9)	3.7 (2.8)	3.5 (2.8)	3.7 (2.8)	0.001
No. of pain sites,† mean (SD)	4.2 (2.1)	3.9 (2.0)	3.7 (1.9)	3.9 (2.0)	<0.001
Pain frequency over previous year,* % (n)					<0.001
Daily	27 (146)	23 (428)	22 (413)	23 (987)	
Over a month	38 (205)	39 (736)	37 (684)	38 (1625)	
8-30 d	23 (120)	27 (502)	25 (457)	25 (1079)	
1-7 d	12 (63)	11 (212)	16 (291)	13 (566)	
15D score,† mean (SD)	0.89 (0.09)	0.92 (0.06)	0.93 (0.05)	0.92 (0.06)	<0.001

N varies in pain dimension, sleeping naps and medication analyses because of missing data.

* χ^2^ test.

† Kruskal–Wallis test.

The mean values of pain intensity, pain-related disability at work, NPS, and daily pain frequency were higher among E-types than in other chronotypes (*P* ≤ 0.001; Table 1). A congruent trend was also seen with respect to the mean of the 15D score: 0.89 (SD: 0.09) for E-types, 0.93 (0.05) for I-types, and 0.92 (0.06) for M-types (*P* < 0.001). The exact 15D scores in each pain dimension across chronotypes are presented in Supplement 2 (available as supplemental digital content at http://links.lww.com/PAIN/B584). For instance, the means of 15D score were 0.93 (SD: 0.07) and 0.82 (0.12) among E-types with 1-site and 8-site pain, respectively, while the corresponding figures were 0.96 (0.04) and 0.90 (0.06) among M-types, respectively. There were only minor differences in the distribution or mean values of sex, sufficiency of sleep duration, pain dimensions, pain medication use, and 15D score between the study sample and nonrespondents (Appendix 1, http://links.lww.com/PAIN/B584).

Table 2 shows the univariate associations of pain dimensions and chronotypes with HRQoL. Each dimension and chronotype were significantly related to HRQoL. As most chronotype × pain interaction terms were statistically significant (*P* < 0.05; Appendix 2, http://links.lww.com/PAIN/B584), we conducted additional analyses, stratified by chronotype, to further demonstrate their role in the pain dimension-HRQoL-associations (Table 3).

**Table 2:** Univariate associations between pain dimensions, chronotypes, and 15D among the Northern Finland Birth Cohort 1966 members with musculoskeletal pain at 46 years.

	N	B coefficient	*P*	95% confidence interval
Pain dimensions				
Intensity	3807	−0.007	<0.001	−0.008 to −0.006
Pain-related disability at work	4027	−0.007	<0.001	−0.007 to −0.006
No. of pain sites	3752	−0.012	<0.001	−0.013 to −0.011
Frequency over previous year				
Daily	987	−0.056	<0.001	−0.063 to −0.050
Over a month	1625	−0.024	<0.001	−0.030 to −0.018
8-30 d	1079	−0.010	0.003	−0.016 to −0.003
1-7 d	566	Ref.		
Chronotypes				
Evening	534	−0.041	<0.001	−0.047 to −0.035
Intermediate	1878	−0.012	<0.001	−0.016 to −0.008
Morning	1845	Ref.		

Interaction terms (chronotype × intensity; chronotype × pain-related disability at work; chronotype × number of pain sites; and chronotype × frequency) were included in the models. Most of them were statistically significant (*P* < 0.05), see Appendix 2 (http://links.lww.com/PAIN/B584).

**Table 3:** Associations between pain dimensions and 15D among the Northern Finland Birth Cohort 1966 members with musculoskeletal pain at 46 years, stratified by chronotype.

	Evening			Intermediate			Morning		
	B	*P*	95% CI	B	*P*	95% CI	B	*P*	95% CI
Intensity									
Model I	**−0.009**	<0.001	−0.012 to −0.006	**−0.007**	<0.001	−0.008 to −0.006	**−0.006**	<0.001	−0.007 to −0.005
Model II	**−0.006**	<0.001	−0.008 to −0.003	**−0.006**	<0.001	−0.007 to −0.005	**−0.004**	<0.001	−0.005 to −0.003
Model III	**−0.005**	<0.001	−0.008 to −0.003	**−0.005**	<0.001	−0.006 to −0.004	**−0.004**	<0.001	−0.005 to −0.003
Pain-related disability at work									
Model I	**−0.009**	<0.001	−0.011 to −0.006	**−0.007**	<0.001	−0.008 to −0.006	**−0.005**	<0.001	−0.006 to −0.004
Model II	**−0.005**	<0.001	−0.008 to −0.003	**−0.005**	<0.001	−0.006 to −0.004	**−0.004**	<0.001	−0.005 to −0.003
Model III	**−0.005**	<0.001	−0.008 to −0.003	**−0.005**	<0.001	−0.006 to −0.004	**−0.004**	<0.001	−0.005 to −0.003
No. of pain sites									
Model I	**−0.015**	<0.001	−0.019 to −0.012	**−0.011**	<0.001	−0.012 to −0.010	**−0.010**	<0.001	−0.011 to −0.008
Model II	**−0.011**	<0.001	−0.014 to −0.008	**−0.008**	<0.001	−0.009 to −0.007	**−0.008**	<0.001	−0.009 to −0.007
Model III	**−0.011**	<0.001	−0.014 to −0.007	**−0.008**	<0.001	−0.009 to −0.006	**−0.007**	<0.001	−0.008 to −0.006
Frequency over previous year									
Model I									
Daily	**−0.078**	<0.001	−0.103 to −0.053	**−0.052**	<0.001	−0.062 to −0.042	**−0.046**	<0.001	−0.054 to −0.039
Over a month	−0.015	0.224	−0.039 to −0.009	**−0.025**	<0.001	−0.034 to −0.015	**−0.022**	<0.001	−0.029 to −0.015
8-30 d	−0.007	0.612	−0.033 to −0.019	−0.009	0.062	−0.019 to 0.000	**−0.008**	0.048	−0.015 to −0.00008
1-7 d	Ref.			Ref.			Ref.		
Model II									
Daily	**−0.045**	<0.001	−0.067 to −0.023	**−0.040**	<0.001	−0.049 to −0.031	**−0.038**	<0.001	−0.045 to −0.030
Over a month	0.002	0.856	−0.019 to 0.023	**−0.019**	<0.001	−0.027 to −0.010	**−0.016**	<0.001	−0.023 to −0.009
8-30 d	0.004	0.740	−0.019 to 0.026	−0.005	0.262	−0.014 to 0.004	−0.005	0.206	−0.012 to 0.003
1-7 d	Ref.			Ref.			Ref.		
Model III									
Daily	**−0.045**	<0.001	−0.067 to −0.022	**−0.039**	<0.001	−0.048 to −0.030	**−0.034**	<0.001	−0.041 to −0.027
Over a month	0.001	0.895	−0.020 to 0.022	**−0.018**	<0.001	−0.026 to −0.010	**−0.015**	<0.001	−0.022 to −0.009
8-30 d	0.003	0.771	−0.019 to 0.026	−0.005	0.240	−0.014 to 0.003	−0.005	0.200	−0.012 to 0.002
1-7 d	Ref.			Ref.			Ref.		

Model I: adjusted for sex. Model II: adjusted for sex, mental distress, and presence of coexisting diseases. Model III: adjusted for sex, mental distress, presence of coexisting diseases, and sufficiency of sleep duration.

Bold values are statistically significant.

CI, confidence interval.

Each pain dimension was related to HRQoL in the chronotype-specific models before and after adjustments (Table 3). In the sex-adjusted models (model I), the reduction of HRQoL seemed to be higher among E-types than among I- or M-types with increasing pain intensity, pain-related disability at work, NPS, and pain frequency. After adjusting for mental distress and presence of coexisting diseases (model II), the reduction of HRQoL attenuated across all pain dimensions but still tended to reduce more among E-types than among M-types. Adjusting further for sufficiency of sleep duration did not significantly change the results (model III). In nearly all models, HRQoL appeared to reduce the least among M-types.

## 4. Discussion

In the birth cohort of 4257 working-aged subjects, the reduction in HRQoL in relation to pain appeared to be more pronounced among E-types than among M-types, with respect to all pain dimensions (pain intensity, pain-related disability at work, NPS, and pain frequency) in the sex-adjusted analyses. After adjustments for all covariates (sex, mental distress, presence of coexisting diseases, and sufficiency of sleep duration), this was particularly seen in terms of NPS and pain frequency. Morning-types with MSK pain tended to report the mildest reductions in HRQoL in comparison to E- and I-types.

Overall, 13% of the study sample were observed to be E-types. A greater number of these reported insufficient sleep duration (25%), severe mental distress (35%), and coexisting diseases (67%) than the other chronotypes. Even though the present study sample was limited to individuals with MSK pain, corresponding findings have also been previously published. Merikanto et al.^35^ found that 13% of Finns represent E-types at the general adult population level. Moreover, insufficient sleep was recorded to be most frequent among E-types and was also reported by 25% of E-types in the general Finnish adult population.^34^ In addition, E-types have been acknowledged to report more mental disorder symptoms and diagnoses, including those severe enough to require hospitalization,^3,7,36,46^ and to also have more, nonmental health–related health complaints in general.^6^ In contrast to expectations, there were no differences in taking naps between the chronotypes, which on the other hand, may be related to indirectly measured data. It is also possible that instead of taking naps, E-types will try to compensate their insufficient sleep by catch-up sleep on free days as indicated in a previous population-based study.^34^

Health-related quality of life is described as a measure of individual estimates regarding a person's current state of health and quality of life and, therefore, provides broad insight into the health-related real-life burden.^14^ Maintaining and improving HRQoL is an essential task of societies and health care professionals worldwide, as with reduced HRQoL, individuals have a greater need for health care services^44^ and have a higher risk of premature mortality.^39^ Earlier studies have recognized several factors affecting HRQoL in MSK pain. These include sex, mood, and sleep.^16^ The present findings have reduced the knowledge gap on the role of chronotypes in HRQoL among subjects with MSK pain. Because the presence of coexisting diseases and mental distress were controlled for in the present analyses, the observed reductions in HRQoL can be mainly attributed to MSK pain itself.

Evening-types seemed to experience a higher reduction in HRQoL than M-types when suffering from pain, as defined by 4 main pain dimensions. In the sex-adjusted analyses, the magnitude of differences in the level of 15D score appeared to exceed the minimum clinically important change (0.015) in increasing pain intensity, pain-related disability at work, NPS, and pain frequency (eg, when subjects had 3-site MSK pain or daily pain). However, after adjusting for mental distress and presence of coexisting diseases, the differences in HRQoL levels narrowed and did not exceed the discrepancy of 0.015 as only in 1 pain dimension analysis. These observations demonstrate that while E- and M-types differ in HRQoL, mental distress and presence of coexisting diseases contribute significantly to the HRQoL, as has been previously suggested.^19,51^ On the other hand, it is worth noting that the relationship between NPS and HRQoL provided clinically relevant differences between chronotypes in the 15D score, even after adjusting for all confounders, including the subject's own estimate regarding sleep sufficiency. Consequently, when considered as a whole, our findings can be viewed as indicative of E-types being particularly vulnerable to the consequences of MSK pain, especially when having multiple pain sites or daily pain.

In their study, Merikanto et al.^33^ showed that E-types are more likely than M-types to seek health care due to MSK pain, giving support to the present inference. Our study findings can be potentially explained in relation to differences in the psychological features discussed below or some underlying biological mechanisms, for instance, related to potential genetic discrepancies between M- and E-types. On the other hand, eveningness associates significantly with disruption in the sleep–wake rhythm, which is a more comprehensive phenomenon than sleep disturbances themselves by virtue of contributing to the body's well-being and recovery at several levels.^6,34^ One potential explanation for the observations may also be related to characteristics of job performed, for example, shift work, because it seems that more E-types than M-types work beyond the day work hour,^41^; E-types appear to especially work during nights (in addition to day work),^41^ which on the other hand may even fit their sleep–wake rhythms better than working daytime. Our results are not likely to be influenced by pain medication use because there were no significant differences between the chronotypes in this regard. Sleep medication was in turn more commonly used among E-types than among M-types, which may explain the higher reduction in HRQoL.

To date, only a few pain studies have examined the relevance of chronotype to HRQoL. In a study on 100 fibromyalgia patients,^48^ E-types were found to report a higher level of reduced quality of life than M-types and had increased symptom severity, in line with the present findings. In turn, Habers et al.^18^ found no significant chronotype differences in HRQoL among 121 rheumatoid arthritis patients. Discrepant age distribution, different methodologies, and smaller samples of individuals with a specific disease known to induce pain may explain the previous, contradictory observations. The present study was based on the general working-age population and focused on MSK pain and its main dimensions, thus providing a wider perspective than previous studies on HRQoL among distinct chronotypes with MSK pain.

Even though the associations between MSK pain and HRQoL was observed irrespective of chronotype, M-types appeared to experience them to the smallest degree. This is likely to mirror the notion that morningness attenuates the negative effects of MSK pain on HRQoL. In the previous literature, morningness has been found to be favorable to mental health,^32^ MSK pain,^31^ and HRQoL in general.^45^ Morning-types also tend to have a healthier lifestyle^38^ and express a higher level of positive psychological features, such as resilience^26^ and self-compassion.^28^ These aspects may influence better overall well-being and better surveillance of pain and, consequently, better HRQoL. Moreover, it may be that the unfavorable fear-avoidance beliefs associated with pain-related consequences are less concentrated among M-types.^47^

The following elements form the strengths of this study. The present study was the first general population study to evaluate the role of chronotypes in MSK pain-HRQoL associations. In addition, the study population (NFBC1966) is a large and an unselected cohort of Northern Finnish adults, improving the generalizability of our results to the Finnish population. Moreover, we had data on all 4 main pain dimensions, enabling a comprehensive exploration of MSK pain.

However, the study also has limitations. Because of the long-term data collection of the original birth cohort, dropouts may have caused selection bias in the data set. However, there were only minor differences in most of the studied variables between the study sample and the nonrespondents. With respect to questions on pain dimensions, it is possible that individuals understood them differently. In addition, as in all questionnaire-based studies, the existence of recall bias cannot be ruled out. On the other hand, there are no validated methodologies for measuring all pain dimensions objectively in as large a sample size as ours. Moreover, as pain always occurs in a biopsychosocial context and is a subjective experience, there have been debates about the advantages of objective pain measurement.^40^ Finally, being a cross-sectional study, the design precludes establishment of cause-and-effect relationships and only enables reporting of associations.

In conclusion, the present findings suggest that eveningness intensifies the associations between MSK pain and HRQoL when compared with morningness and that chronotype should be accounted for in MSK treatment and rehabilitation measures aimed at maintaining or improving HRQoL. Such measures should especially target E-types with MSK pain. In a wider point of view, chronotype might hold potential to be used as one of the prognostic factors in the selection of stratified care in individuals with MSK pain.^10,13^ We encourage future studies, firstly to confirm our findings in other cohorts and ,secondly, to clarify the potentially explanatory pathways behind HRQoL discrepancies between E- and M-types with MSK pain.

## Conflict of interest statement

The authors have no conflicts of interest to declare.

## Appendix A. Supplemental digital content

Supplemental digital content associated with this article can be found online at http://links.lww.com/PAIN/B584.

## Supplementary Material

SUPPLEMENTARY MATERIAL
