# Supplementary material for: Eveningness intensifies the association between musculoskeletal pain and health-related quality of life: a Northern Finland Birth Cohort Study 1966
Source: Pain. 2022 Feb 7;163(11):2154–61. doi: 10.1097/j.pain.0000000000002609 (PMC9578528; doi:10.1097/j.pain.0000000000002609)
Supplement: SUPPLEMENTARY MATERIAL [file jop-163-02154-s001.pdf]

Supplement 1. Included diseases.

Hypertension
Heart failure
Type 2 diabetes
Hypothyroidism
Hyperthyroidism
Inflammatory bowel disease
Psoriasis
Hand eczema
Other skin disease
Ocular hypertension
Glaucoma
Macular degeneration
Epilepsy
Migraine
Cerebrovascular disease
Other disease in the nervous system
Cancer (other but skin cancer)
Alcohol dependence disorder
Other substance use disorder
Sleep apnea

Supplement 2. Mean values and standard deviation (SD) of 15D across pain dimensions among the Northern Finland Birth Cohort 1966 members at 46 years, stratified by chronotype.

	Evening		Intermediate		Morning	
	N	Mean (SD)	N	Mean (SD)	N	Mean (SD)
Intensity						
**0**	26	0.92 (0.12)	121	0.95 (0.05)	200	0.96 (0.04)
**1**	34	0.91 (0.07)	120	0.95 (0.04)	133	0.96 (0.04)
**2**	63	0.93 (0.05)	245	0.94 (0.05)	194	0.95 (0.04)
**3**	70	0.92 (0.06)	265	0.93 (0.06)	253	0.93 (0.05)
**4**	49	0.90 (0.07)	178	0.93 (0.06)	179	0.93 (0.06)
**5**	71	0.90 (0.08)	208	0.92 (0.06)	208	0.93 (0.06)
**6**	37	0.87 (0.09)	184	0.91 (0.07)	140	0.93 (0.05)
**7**	54	0.86 (0.11)	169	0.89 (0.07)	124	0.92 (0.06)
**8**	45	0.88 (0.09)	129	0.90 (0.06)	128	0.91 (0.06)
**9**	16	0.88 (0.10)	56	0.89 (0.08)	58	0.90 (0.07)
**10**	11	0.82 (0.14)	20	0.89 (0.09)	19	0.92 (0.07)
**Total**	476		1695		1636	
Pain-related disability at work						
**0**	61	0.89 (0.11)	254	0.95 (0.05)	320	0.96 (0.04)
**1**	72	0.93 (0.07)	226	0.94 (0.05)	208	0.95 (0.04)
**2**	56	0.93 (0.05)	250	0.94 (0.05)	243	0.94 (0.04)
**3**	61	0.91 (0.06)	230	0.93 (0.06)	198	0.93 (0.06)
**4**	38	0.90 (0.06)	158	0.92 (0.06)	150	0.93 (0.05)
**5**	50	0.89 (0.07)	177	0.92 (0.05)	166	0.92 (0.06)
**6**	40	0.89 (0.08)	129	0.90 (0.07)	115	0.93 (0.05)
**7**	40	0.87 (0.10)	157	0.90 (0.06)	109	0.92 (0.06)
**8**	41	0.84 (0.10)	118	0.89 (0.08)	127	0.91 (0.06)
**9**	23	0.87 (0.10)	66	0.90 (0.07)	64	0.90 (0.07)
**10**	17	0.81 (0.15)	33	0.87 (0.10)	30	0.92 (0.06)
**Total**	499		1798		1730	
Number of pain sites						
**1**	45	0.93 (0.07)	169	0.95 (0.05)	228	0.96 (0.04)
**2**	72	0.93 (0.06)	296	0.94 (0.05)	271	0.95 (0.05)
**3**	78	0.91 (0.08)	321	0.94 (0.05)	324	0.94 (0.05)
**4**	84	0.90 (0.09)	259	0.92 (0.06)	286	0.94 (0.05)
**5**	68	0.88 (0.08)	214	0.91 (0.07)	237	0.91 (0.06)
**6**	44	0.90 (0.06)	168	0.91 (0.06)	116	0.91 (0.06)
**7**	43	0.82 (0.11)	103	0.89 (0.06)	90	0.91 (0.06)
**8**	40	0.82 (0.12)	111	0.88 (0.08)	85	0.90 (0.06)
**Total**	474		1641		1637	
Frequency over previous year						
**Daily**	148	0.84 (0.10)	428	0.89 (0.07)	413	0.91 (0.06)
**Over a month**	205	0.91 (0.08)	736	0.92 (0.06)	684	0.93 (0.05)
**8–30 days**	120	0.92 (0.07)	502	0.94 (0.05)	457	0.95 (0.04)
**1–7 days**	63	0.92 (0.09)	212	0.95 (0.05)	291	0.96 (0.05)
**Total**	534		1878		1845	

Appendix 1. Representativeness of study sample at 46 years (n=4257).

**Variables**	Respondents % (n)	Non-respondents % (n)	P value
*Sex**, % (n)			<0.001
Men	42 (1785)	52 (1514)	
Women	58 (2472)	48 (1376)	
*Sufficiency of sleep duration**, % (n)			<0.001
Insufficient	9 (396)	9 (211)	
Somewhat insufficient	48 (2023)	43 (1019)	
Sufficient	43 (1838)	48 (1159)	
*Sleeping naps**, % (n)			0.062
No	61 (2409)	59 (1318)	
Yes	39 (1510)	41 (914)	
*Mental distress**, % (n)			0.940
Severe	20 (863)	20 (439)	
Mild	80 (3394)	80 (1718)	
*Presence of co-existing diseases**, % (n)			0.251
Yes	57 (2433)	56 (1425)	
No	43 (1824)	44 (1132)	
*Using pain medication**, % (n)			<0.001
Yes	68 (2365)	61 (1166)	
No	32 (1119)	39 (734)	
*Using sleep medication**, % (n)			0.292
Yes	4 (142)	5 (89)	
No	96 (3342)	95 (1811)	
Chronotypes			0.167
Evening	13 (534)	11 (234)	
Intermediate	44 (1878)	43 (884)	
Morning	43 (1845)	46 (938)	
*Pain intensity***, mean (SD)	4.1 (2.6)	3.4 (2.9)	<0.001
*Pain-related disability at work***, mean (SD)	3.7 (2.8)	3.1 (3.0)	<0.001
*Number of pain sites***, mean (SD)	3.9 (2.0)	3.2 (2.4)	<0.001

*Pain frequency over previous year**, % (n)			<0.001
Daily	23 (987)	26 (515)	
Over a month	38 (1625)	33 (653)	
8–30 days	25 (1079)	26 (514)	
1–7 days	13 (566)	15 (305)	
*15D score***, mean (SD)	0.92 (0.06)	0.93 (0.07)	<0.001

*x^2 test
**Kruskal-Wallis test
N varies in pain dimension, sleeping naps and medication analyses due to missing data.
SD=standard deviation.

Appendix 2. Interaction terms for chronotype and pain dimensions.

**Interaction terms**	B coefficient	P value	95% Confidence interval
*Chronotype*intensity*			
Evening*intensity	-0.003	0.006	-0.006 to -0.001
Intermediate*intensity	-0.002	0.029	-0.003 to -0.000
Morning*intensity	Ref.		
*Chronotype*pain-related disability at work*			
Evening*pain-related disability at work	-0.003	0.002	-0.005 to -0.001
Intermediate*pain-related disability at work	-0.002	0.009	-0.003 to -0.000
Morning*pain-related disability at work	Ref.		
*Chronotype*number of pain sites*			
Evening*number of pain sites	-0.005	<0.001	-0.008 to -0.002
Intermediate*number of pain sites	-0.001	0.194	-0.003 to 0.001
Morning*number of pain sites	Ref.		
*Chronotype*frequency*			
Evening*daily pain	-0.030	0.003	-0.050 to -0.010
Intermediate*daily pain	-0.006	0.357	-0.020 to 0.007
Morning*daily pain	Ref.		
Evening*over a month	-0.009	0.380	-0.011 to 0.028
Intermediate* over a month	-0.003	0.627	-0.016 to 0.009
Morning* over a month	Ref.		
Evening*8–30 days	-0.002	0.850	-0.019 to 0.023
Intermediate*8–30 days	-0.002	0.779	-0.015 to 0.011
Morning*8–30 days	Ref.		
